# An adaptable and personalized framework for top-N course recommendations in online learning

**DOI:** 10.1038/s41598-024-56497-1

**Published:** 2024-05-06

**Authors:** Samina Amin, M. Irfan Uddin, Ala Abdulsalam Alarood, Wali Khan Mashwani, Ahmed Omar Alzahrani, Hamdan Ahmed Alzahrani

**Affiliations:** 1 Institute of Computing, Kohat University of Science and Technology (KUST), Kohat, 26000 Pakistan; 2 College of Computer Science and Engineering, University of Jeddah, Jeddah, Saudi Arabia; 3 Institute of Numerical Sciences, Kohat University of Science and Technology (KUST), Kohat, 26000 Pakistan; 4 Faculty of Computer Science and Engineering, University of Jeddah, Jeddah, Saudi Arabia; 5 College of Computing and Informatics, Saudi Electronic University, Makkah, Saudi Arabia

**Keywords:** Reinforcement learning, Deep reinforcement learning, Online learning, e-learning, MOOC, Recommender system, Computational science, Computer science, Information technology, Scientific data

## Abstract

In recent years, the proliferation of Massive Open Online Courses (MOOC) platforms on a global scale has been remarkable. Learners can now meet their learning demands with the help of MOOC. However, learners might not understand the course material well if they have access to a lot of information due to their inadequate expertise and cognitive ability. Personalized Recommender Systems (RSs), a cutting-edge technology, can assist in addressing this issue. It greatly increases resource acquisition through personalized availability for various people of all ages. Intelligent learning methods, such as machine learning and Reinforcement Learning (RL) can be used in RS challenges. However, machine learning needs supervised data and classical RL is not suitable for multi-task recommendations in online learning platforms. To address these challenges, the proposed framework integrates a Deep Reinforcement Learning (DRL) and multi-agent approach. This adaptive system personalizes the learning experience by considering key factors such as learner sentiments, learning style, preferences, competency, and adaptive difficulty levels. We formulate the interactive RS problem using a DRL-based Actor-Critic model named DRR, treating recommendations as a sequential decision-making process. The DRR enables the system to provide top-N course recommendations and personalized learning paths, enriching the student's experience. Extensive experiments on a MOOC dataset such as the 100 K Coursera course review validate the proposed DRR model, demonstrating its superiority over baseline models in major evaluation metrics for long-term recommendations. The outcomes of this research contribute to the field of e-learning technology, guiding the design and implementation of course RSs, to facilitate personalized and relevant recommendations for online learning students.

## Introduction

With the advancement of information technology, online learning has rapidly become a necessary platform for knowledge acquisition. Massive Open Online Courses (MOOC) platforms are recent advancements of this online learning movement. They have received a lot of attention from the academic and public domains. This development of numerous MOOC platforms, including Udemy, edX, Coursera, Udacity, and others. More than 100 million students worldwide have access to convenient education through these platforms. As they offer an affordable way to take appropriate courses at many prestigious universities^1^. Due to COVID-19's influence in the past two years, 2021 has witnessed a rise in MOOC, making it more challenging to execute traditional teaching methods in many regions^1^. MOOC has quickly replaced the traditional mode of classroom learning. It enabled the idea of being able to study anywhere and whenever possible. Learners frequently use online learning platforms to increase their knowledge, develop new abilities or skills, and conduct academic research.

Despite the many advantages that come with MOOC, it also leads to the extremely key challenge of information overload^1^. In order to assist learners in choosing appropriate courses from the thousands of courses accessible for learning, it is also required to consider the learner's career decisions and other factors. To address this, personalized Recommender Systems (RSs)^2,3^ have emerged as a solution, aiming to match learners' preferences with relevant content and mitigate information overload.

While existing RSs have employed techniques like content-based filtering, Collaborative Filtering (CF), and hybrid methods^4,5^. Recently, motivated by the quick development of online learning, various recommendation methods have been developed^6,7^. They applied supervised learning and data mining techniques to develop RSs for MOOC. However, these existing RSs usually neglect interactions between a user and the recommendation algorithm. They often fall short in capturing user interactions and adapting to new circumstances, which leads to poor recommendation outcomes.

The nature of learner interaction with RSs turns the recommendation problem into not just a classification/prediction issue but also a sequential decision problem. The machine learning domain focuses on how intelligent agents communicate with their surroundings^6,7^. Whereas RL^8^ proposes methods for modeling user-agent interactions. It adopts the policy through trial-and-error search, which is helpful for sequential decision-making. Due to the evaluation of overall actions, the conventional RL approach has the drawback of potentially being inefficient if the action space is too big^9^. While DRL^10,11^ offers more advanced learning algorithms and function approximation features to tackle challenges in artificial intelligence. However, it has been applied in many different applications; e.g., games^12^, trading^13^, robotics^14,15^, disruption risk identification^16^, and the Internet of Things (IoT)^17–20^. Recently, a new research trend in recommendation research is the use of RL to address recommendation problems^4^. In practice, RL-based RSs have been used for a variety of specialized applications, including movie recommendations^21^, news recommendations^22^, treatment recommendations^23,24^, opinion analysis^25^, and e-learning^26,27^.

Nowadays, the majority of students conduct their studies online. Students are having a very hard time picking appropriate content to come to this online learning system. Since the internet has a massive, varied, and dynamic data collection. A study of the user's profile and interests should be used to develop a personalized RS for the learners. In MOOC platforms, the learner’s browsing pattern, efficiency, and domain relevancy should all be acquired by intelligent agents; e.g., the way learners navigate varies from one another. The RL agent can learn from dynamically changing browsing activity and then make recommendations to the learner. The learner's feedback is collected by the agent. The agents should dynamically modify information based on the user's preferences as well as from time to time in online course recommendations. Due to the passage of time, one piece of information becomes outdated. MOOC systems should dynamically update content and topics. Course content should be updated regularly based on learner feedback and temporal variations. To address the drawbacks, it is necessary to create a multi-agent-based intelligent learning environment that automatically tracks the factors, which influence learning in various contexts and then updates recommended learning paths and content from one learner to the next.

To the best of our knowledge, none of the current studies have applied multi-agent DRL techniques for MOOC RS^10^. The advances in RL have had phenomenal achievements across many domains. Although the multi-agent domain has been dominated by its single-agent counterpart throughout this development, multi-agent RL is making headway quickly, and the most recent achievements deal with issues that are challenging in real-world applications. Inspired by recent advancements in AI and DRL, in this work, a novel multi-agent DRL intelligent framework is proposed for personalized learning and an optimizing RS for multipath navigation adaptively in MOOC. Another challenge is to analyze how the agents learn by themselves and select optimal actions according to deviations in their environment. If any agent fully grasps the policy, it will be able to decide what is the best approach for action in any given state. For example, if the Agent has acquired the best policy, it can accurately determine whether a relevant course is present to be recommended to the learner. Even humans do not understand how to recommend suitable course content; we simply recognize them when we see them. This makes the task difficult for a machine to complete and unfeasible when performed with state-of-the-art methods. These challenges will be framed within the Markov Decision Process (MDP) framework^8^. Then, the interactive RS problem will be formulated using a DRL recommendation framework such as an Actor-Critic named DRR.

The prime contributions and technical developments of the proposed approach are outlined as follows.

1. To propose an intelligent agent based on DRR such as Actor-Critic for top-N course recommendations in MOOC.
2. To develop an effective technique for the proposed smart learning environment to encourage students to benefit from the learning materials and become independent learners.
3. To accommodate potential dynamic changes in students' states and learning preference levels, by leveraging MDP terminologies where the agent is solely concerned with the present state of the process and lacks curiosity about the entire historical context or previous records when a new learner is registered, or new information is added to the system.
4. To evaluate the proposed model, experiments are conducted through simulations and compare its performance with current state-of-the-art solutions in sequential recommendation tasks for optimal results.
5. The experimental findings verify the effectiveness of the proposed model in providing top-N course recommendations in MOOCs by computing evaluation metrics.

The rest of this article is categorized as follows: Literature review based on traditional RS, RL, and DRL will be discussed in “Literature review”. “Research gap” presents research gaps. A detailed methodology of the proposed framework will be demonstrated in “Overall study framework”. Experimental results and analysis will be presented in “Experiments”. Finally, the conclusions, limitations, and future work are presented in “Conclusions”.

## Literature review

Researchers have done significant work in RSs analysis and provided effective solutions for the recommendation of items using supervised learning and RL methods^4^. Shin et al.,^2^ focused on introducing an RS that can compute the optimal number and schedule of examinations for every learner based on the RL method. Wacharawan et al.^27^ developed an RL-based online recommendation approach. The authors designed an RL agent based on state-action-reward-state-action performing dynamically and continuously in action space. To determine optimal policy, the agent selects the collection of actions. Then the performance of their model is evaluated with the real dataset from an OL system. However, the predictions’ accuracy still needs improvement, as the square root of the mean squared error is relatively high. Along with the approach to determine the ideal values of greedy, learning rate, and discount rate, it is important to investigate the other RL methods. Similarly, Lalitha et al.,^7^ proposed an agent-based recommendation for e-learning by utilizing machine learning and knowledge discovery techniques. In other related work, Zheng et al.,^22^ applied a DRL-based model for news recommendation, while Esfahaani et al., proposed RS for online advertising with the help of DRL. Using RL techniques, some other research studies have been carried out on movie recommendations^21,28^. To perform recommendations, they created an RL-based single agent, but the models are unable to adapt multiple users appropriately if different patterns are observed for distinct user groups. Since they experience fundamental problems while deploying a single agent to carry out many tasks, such as feature space. Employing multiple agents to execute similar tasks in parallel is an alternate approach to improving learning performance. As a result, to make the recommendations more accurate, the existing studies need to be extended.

Moreover, Lin et al.^29^ developed a hierarchical RL with a dynamic recurrent mechanism to provide individualized course recommendations. They suggested a policy gradient method to solve the trade-off between exploration and exploitation while building user profiles. While applying a dynamic baseline to investigate the user’s future preferences, it implemented a recurrent approach through context-aware learning to use the present information. Vedavathi et al.,^30^ proposed a hybrid Elman similarity-based e-learning course RS framework using sentiment analysis. Using similarity measures, their proposed model was utilized to classify the sentiment. Yuan et al.,^31^ designed MOOC recommendations for personalized course RS based on integrating multi-granularity sessions and multi-type interests. Furthermore, by combining network structured features with graph neural networks and user interactive activities with tensor factorization. Zhu et al.,^32^ created a hybrid RS approach. First, a network with a graph structure for teaching assessment was suggested to characterize students, courses, and other entities utilizing the ratings, commentary text, grades, and personal connections of the students. The vectorized representation of the students was then produced by a neural network built on a random walk algorithm after each student learned their unique relational pattern. Finally, a Bayesian probabilistic-based tensor factorization is used to learn and predict students’ ratings for lectures they have not yet attended. This is done by identifying these personalization features as the third dimension of the rating tensor.

Likewise, Campos et al.,^33^ created RS for MOOC ecosystems, suggesting sections of courses from various MOOC platforms (such as Khan Academy, Udemy, and edX). In order to balance the ecological environment and strengthen connections, their suggested model relied on the student profile and the MOOC ecosystems approach. The results of an experiment were carried out using a dataset with 19 students, three MOOC platforms showed that the adopted methods are more reliable than other methods. Furthermore, it was determined that their recommendations are 62,24% correct, 68.89% valuable, 72.81% dependable, and present new content in 99.12% of cases. These outcomes demonstrate that the strategy helps students close their knowledge gaps. Through the application of machine learning techniques, Nilashi et al.^34^ suggested an RS to promote courses in MOOCs based on the preferences and behavior of students. The approach was created employing multi-criteria ratings taken from learners’ reviews posted online. For text mining, they utilized Latent Dirichlet Allocation; for decision rule creation, they employed decision trees; for learner’s reviews of courses, they applied a self-organizing map; and for preference prediction, they adopted a fuzzy rule-based approach. They also used a feature selection mechanism to pick the most crucial factors for predicting learners’ preferences. The approach was assessed using data gathered from MOOC platforms such as Udemy. The findings demonstrated that the approach is capable of accurately offering learners relevant courses that are catered to their interests.

Symeonidis and Malakoudis^35^ built an xSVD++-based RS model. The “x” stands for a multi-dimensional matrix factorization structure combined with the CF method, which utilizes data from external sources (such as users’ skills, course features, etc.) to forecast course patterns and carry out rating predictions in accordance with them. Additionally, Boratto et al.,^36^ explored how RS works in the context of MOOC, going beyond prediction accuracy. While Lin et al.,^37^ proposed a novel course RS based on dynamic attention mechanism and hierarchical RL, to increase the adaptivity of the RS. J. Using a graph neural network, Wang et al.,^38^ designed a Top-N personalized RS in the MOOC by exploring two different aggregate functions to compact with the learners’ sequence neighbors and then apply an attention algorithm to produce the final course suggestions. Yuanguo^39^ designed a hierarchical and recurrent RL-based context-aware RL technique. Their model can effectively rebuild user profiles for course recommendation. Madani et al.,^40^ developed RS for directing learners into appropriate courses. The suggested approach relies on CF (using the concepts of sentiment analysis) and social filtering to determine the optimal manner for the student to study and to suggest courses that complement their profile and social media material.

## Research gap

The existing landscape of RSs reveals several gaps that hinder their effectiveness. Firstly, prevalent RSs commonly adopt static techniques for recommendations, lacking an understanding that recommendation actions are sequential decision-making problems. This oversight contributes to shortcomings in adapting to new circumstances and addressing the cold-start problem. Additionally, these conventional RSs frequently overlook user interactions with the recommendation algorithm. This leads to a failure in a timely recording of user feedback, resulting in suboptimal recommendations. Furthermore, existing methods, while competent in classifying learners’ intelligence levels, often fall short in regulating actions for specific topics. The reliance on RL-based single agents in these methods becomes problematic when adapting to distinct user groups with varying patterns.

While these techniques are competent in terms of recommendations to some point; however, they have the flaw of ignoring the learner’s shifting preferences in personalized adaptive sequential learning ability/activities. Furthermore, these methods may not precisely capture the learner’s evolving preferences for individual content, especially when the learners’ preferences are being changed over time across various courses over time, which results in inadequate recommendations in MOOC scenarios.

To address these gaps, this study proposes a novel multi-agent DRL framework for top-N MOOC recommendations. The proposed framework aims to optimize RS for adaptive multipath navigation in the MOOC environment. The primary contribution lies in exploring how agents, through DRL, can maximize learner satisfaction and minimize interactions to create personalized learning paths. In addition, the proposed work studies the challenge of understanding how agents autonomously learn and select optimal actions based on environmental deviations. By empowering agents to grasp policies, the proposed framework seeks to enable them to make informed decisions for optimal actions in any given state.

## Overall study framework

In this section, the proposed model based on DRR is introduced, which is a top-n list-wise model for implicit feedback. First, the problem formulation is discussed. Then, the proposed model is presented in detail. Finally, it is proved that the discussed models have the potential to be streamlined into a top-N coarse recommendations model, by achieving promising results in terms of recommendation accuracies.

### Problem formulation

The proposed framework can be divided into two main components: the learner agent and the recommendation agent. The learner agent is responsible for modelling the learner's behaviors, learning style, preferences, competency, adaptive difficulty levels, and knowledge level over time. It uses DRL algorithms with MDP terminologies to learn from the learner's interactions with the system and adapt to their changing needs or preferences during the study. The recommendation agent uses the information learned by the learner agent to recommend top-N course content to a learner based on their preferences, adaptive difficulty levels, behavior, knowledge level, etc.

The proposed DRR framework for personalized learning and top-N course recommendation can be seen in Fig. 2. The environment is designed by learners and course material and the roles of the agents are handled by recommendation algorithms. Here, the state is shown as a feature depiction for learners, and action is defined as a feature depiction of course content. When a student wants a course, the agents are given a state representation (i.e., learner's features) and a collection of action representations (i.e., the course competitors' features). The agents will choose the best course of action (e.g., recommend a list of course content to a learner) and reward the learner with feedback. The reward is made up of click labels and an estimate of the learner's activity level. All these recommendations and feedback reports will be saved in the agents' database (memory). The agent will use the data in memory to update its recommendation algorithm at every iteration. This problem can be formulated as follows:

***Agent ***$\left(\mathcal{A}{\varvec{g}}\right)$**:** A program (algorithm) that decides what to display next in a collection of MOOC.

***Environment ***$\left({\varvec{E}}\right)$: The learning framework.

***Action ***$\left(\mathcal{A}\right)$**:** action being the next suitable course to be recommended or recommending a new course. Or it is a rating score vector. The action is a perfect course content that the learner would enjoy studying/reading.

***State ***$\left(\mathcal{S}\right)$**:** A learner's interaction features are depicted as a state. In other words, it is considered as the positive interaction records of the learners. The *state-value* ${\mathbbm{v}} \left(\mathcal{S}\right)$ can be defined to evaluate the goodness of the current state as shown in Eq. (1).

1 $${\mathbbm{v}} \left(\mathcal{S}\right)= {\mathbb{E}} \left\{\mathcal{Q} \left(\mathcal{S}, \partial \right)\right\}$$

***State Transitions*** ${\mathcal{P}}({s}_{\mathcal{T}}+1 \left| {s}_{\mathcal{T}}, {\partial }_{\mathcal{T}}\right)$: The state is modelled as a depiction of a learner's interactions history. As a result, once the learner's response has been gathered, the *state transition* can be determined at an *action* $\partial$ in *state* $\mathcal{S}$ at time $\mathcal{T}$ to ${s}_{\mathcal{T}+1}$ at $\mathcal{T}+1$, as follows in Eq. (2).

2 $${\mathcal{P} }\left({s}_{\mathcal{T}}+{\partial }_{\mathcal{T}} \right|{ s}_{\mathcal{T}} {\partial }_{\mathcal{T}},\dots \dots \dots \dots \dots ,{s}_{\mathcal{T}}, {\partial }_{\mathcal{T}}a1) = \mathcal{P} ({s}_{\mathcal{T}}+1 \left| {s}_{\mathcal{T}}, {\partial }_{\mathcal{T}}\right)$$

***Reward*** ${\mathcal{R}}\left({s}_{\mathcal{T}+1}+{\partial }_{\mathcal{T}}, {s}_{\mathcal{T}}, {\partial }_{\mathcal{T}}\right)$: to be the user satisfaction/ conversion or review. For instance, the reward will be positive if the learner is satisfied with the course and rates it as approximately 4; the reward is more positive if the learner rated the course as 5; if the learner becomes bored, the result will be negative and course rating can be as below 2. Furthermore, in response to the recommendation based on the action and the learner states s, the learner will give their input by clicking, not clicking, rating the course, etc. Following the learner's response, the recommender is given an immediate reward, R(s, a). The *immediate reward* for the agent transmitting from $s {\text{to}} {s}_{\mathcal{T}+1}$ is generated by this function. The *expectation* of *cumulative future reward* will be maximized via the following formula (Eq. 3).

3 $${\mathbb{E}}=\sum_{\mathcalligra{n}=1}^{\mathcal{N}}\stackrel{'}\gamma \mathcal{R}\left( {s}_{\mathcal{T}}+ {s}_{\mathcal{T}+1}\right)$$

**Discount rate ϒ**: used to calculate the current value of future rewards or long-term rewards, which ranges from 0 to 1.

**Figure 1:**
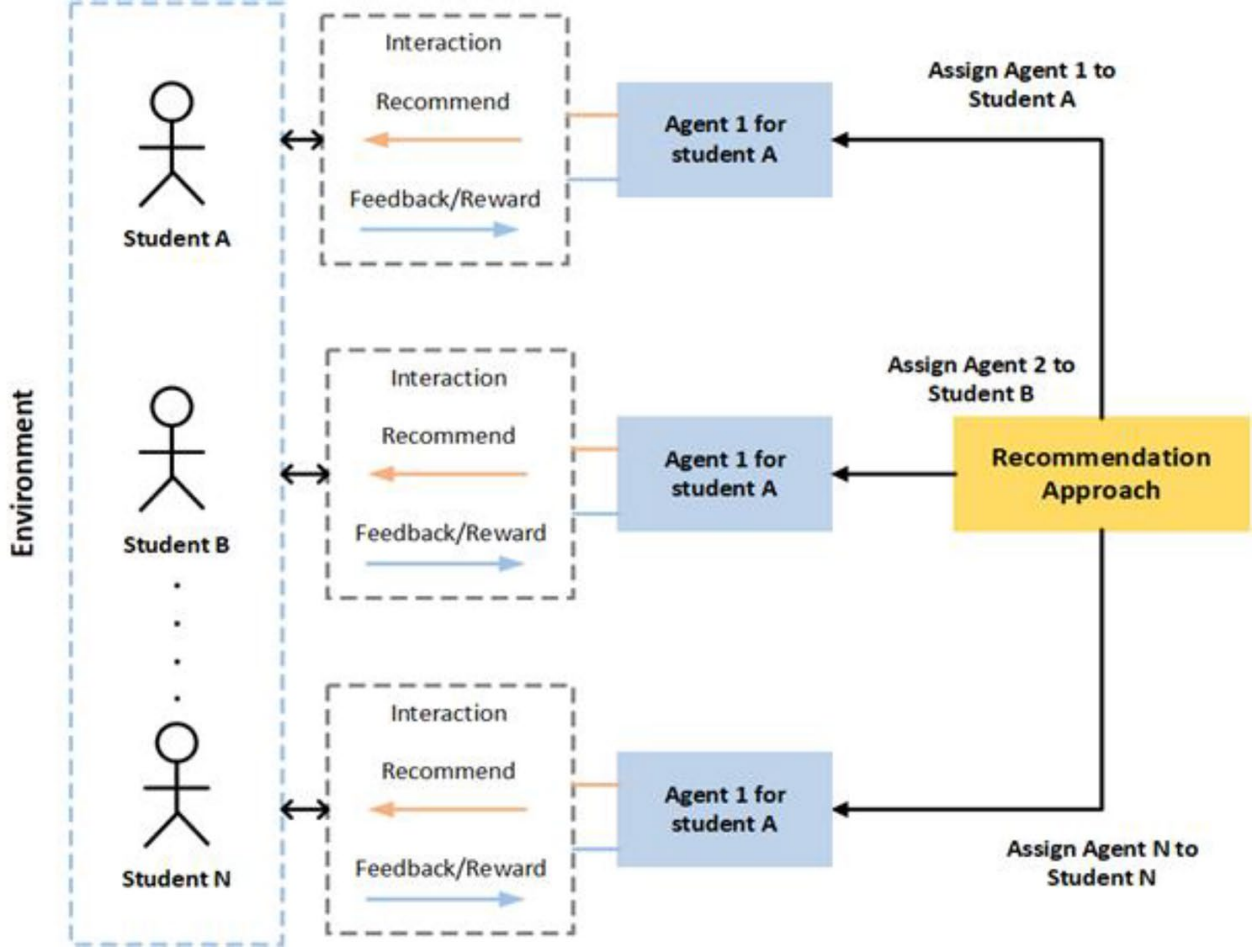
Student-agent interaction for top-N course RS.

For example, Fig. 1 shows recommendation processes in RL, during the student-agent interaction, the model will assign an agent to each student. An agent will recommend the content to the student and the student will give feedback to the agent.

### Proposed DRR model

**Figure 2:**
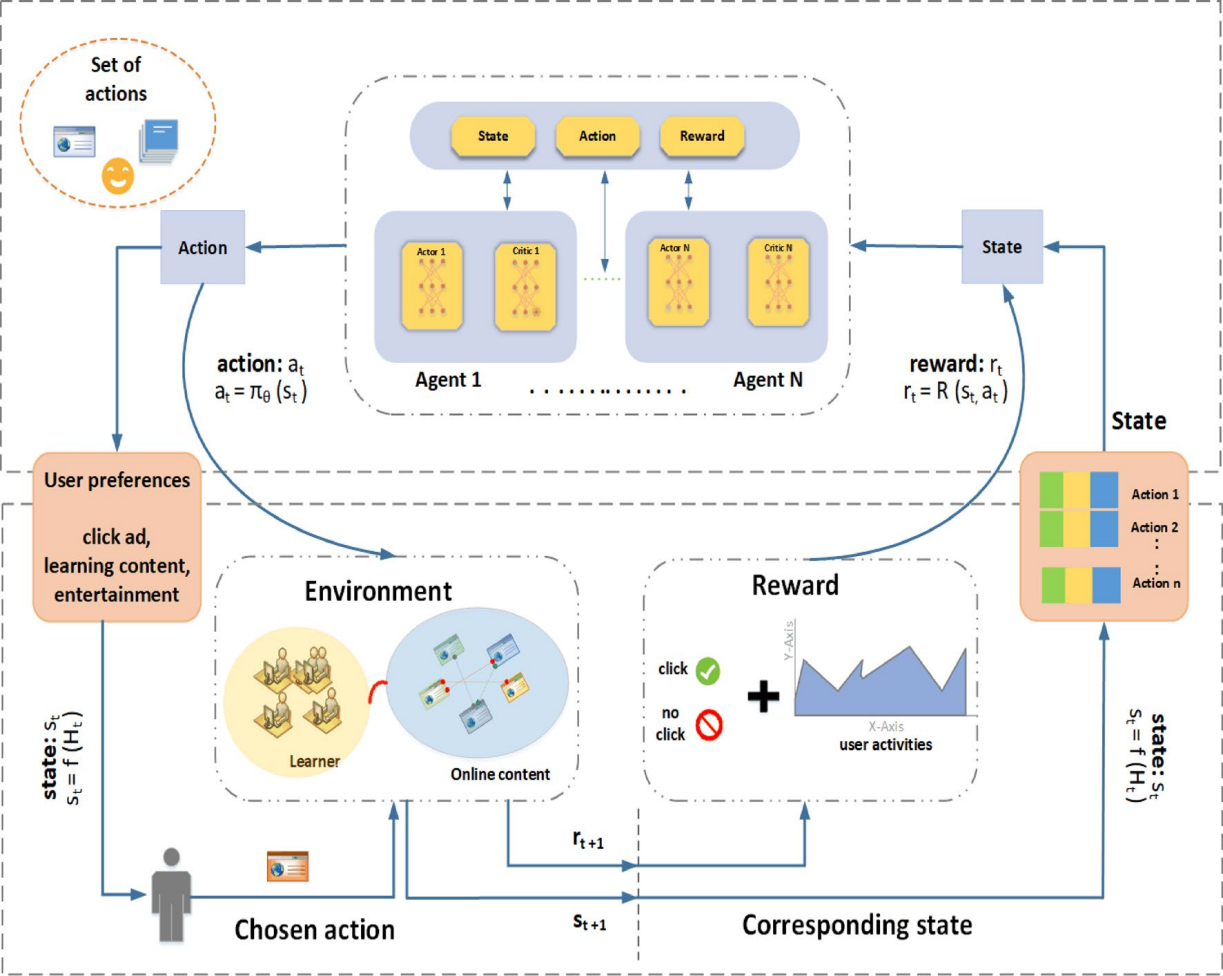
A proposed adaptable and personalized framework for top-N course recommendations in online learning.

This study aims to propose an effective top-N course recommendation framework for RL-based RS. The suggested framework employs many strategies to construct a reliable RS model. To achieve this goal, the interactive RS problem will be formulated using a DRL recommendation framework such as an Actor-Critic named DRR. In this framework, various state representation schemes for training the recommendation policy (actor) and value function (critic) are briefly discussed. The proposed DRR framework for top-N course recommendation can be seen in Fig. 2. Moreover, to remove the noise in the MOOC data, pre-processing is first carried out. The required features are then extracted utilizing the improved TFIDF of feature extraction. To provide robust course recommendations, the DRR-based recommendation agents (Actor-Critic) are designed as follows.

*Actor-Critic* is a DRL-based model that combines elements of value-based and policy-based mechanisms. In this instance, the Actor oversees formulating recommendations (policy-based), and the Critic assesses the effectiveness of the recommendations (value-based). Sequential recommendations can be made using the Actor-Critic model, in which the Actor comes up with the suggestions and the Critic evaluates them. To enhance the suggestions over time, the model is updated using the value function and the policy gradient.

To train the model, we used 100 K Coursera course review data consisting of learners’ reviews for a specific course and their ratings. This data is then input into the Actor Network, which is responsible for making decisions about what course content to recommend next. The actor network generates an ideal course embedding, representing the desired content. To find similarities, these embeddings can be compared to those of other courses. The course material that best suits the student’s needs can then be suggested to them. The Critic Network is essential to this process. It evaluates each decision the Actor Network makes and directs it to find any possible flaws. While the Critic Network helps forecast how learners will behave in the future. For example, if RS recommends that a student read a course material or complete an assignment, and the student complies, they will instantly receive 100. But the student might get bored or want to use social media instead, which would result in a -100 penalty. This is where the Q-Network (Critic) becomes crucial: considering all potential future actions is essential.

#### Proposed architecture

The actor and critic are the two layers that design the network. Here the Critic uses a Q-learning mechanism and is primarily concerned with rewards and the Actor focuses on learning policies, which are the probabilities of choosing the next action. The process begins with course embeddings being directed into the Actor’s state representation module, where these embeddings are transformed into encoded representations. Subsequently, an action is formulated in the form of a vector. This action is then combined with course embeddings, resulting in a concatenated input that is fed into the Critic Module. The main objective of the Critic architecture is to analyze and predict the potential of the upcoming reward. First, a substantial amount of course embeddings are provided to the actor’s state representation module, where they are encoded. Then, a decision is taken (resulting in an action represented by a vector). The action is passed into the Critic Module along with item embeddings to estimate how excellent the reward will be.

In conclusion, this framework enhances the recommendation process by using a two-layered architecture. The Actor layer is responsible for learning the likelihood of selecting specific actions, while the Critic layer is dedicated to assessing the potential rewards. Through a combination of reward evaluations and learned policies, the proposed framework maximizes the decision-making process for personalized recommendations.

The design of the proposed framework can make an adaptable and personalized class environment more valuable. With the help of recommendations, the learner can benefit from more effective learning, and the system can benefit from more effective advertising.

## Experiments

This section outlines the experimental procedure used in this study to evaluate the effectiveness of the DRR model (Actor-Critic), including the dataset description, parameter setting, evaluation metrics, and findings.

First, the dataset is introduced and employed in the experiments. Then, the baseline models are discussed and compared with the proposed models and the metrics that are adopted for evaluation.

### Data description

We first briefly introduce the MOOC dataset that is applied:

**100 K Coursera’s Course Reviews**: to train the proposed models, a 100 K Coursera’s Course Reviews dataset was extracted from the publicly accessible Kaggle repository^41^. This data contains around 120k reviews from learners on Coursera’s MOOC for multiple courses. Each student has an integer rate between [1, 5]. The courses are rated based on the learner’s sentiments, learning style, preferences, competency, and adaptive difficulty levels, the student may have. Table 1 represents an example of the course review dataset that is rated based on these factors.

**Figure 3:**
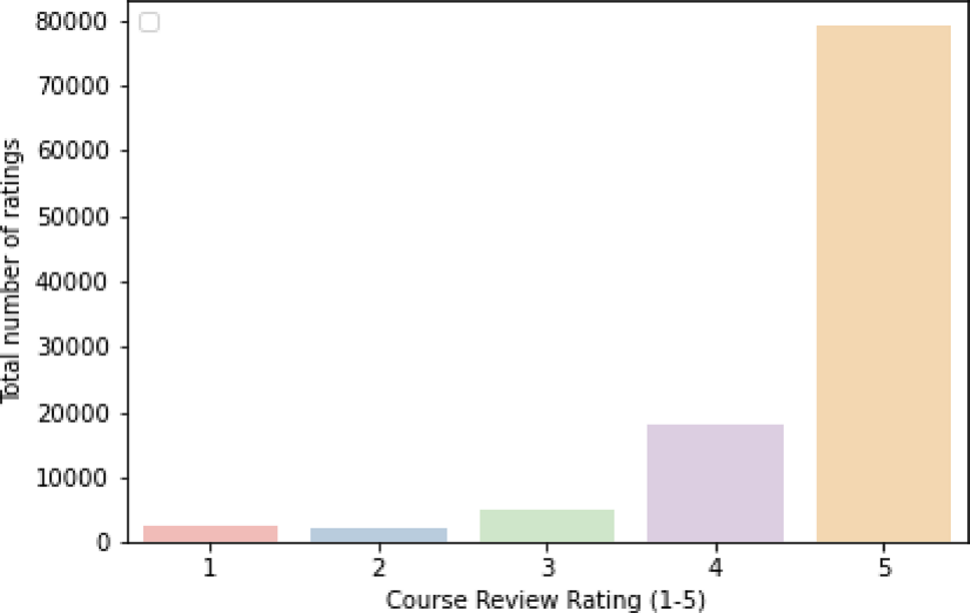
Distribution of Coursera course review into five ratings; rate 5 has a total of 79,173 ratings;4 (18,054); 3 (5071); 1 (2469) and 2 (2251).

**Figure 4:**
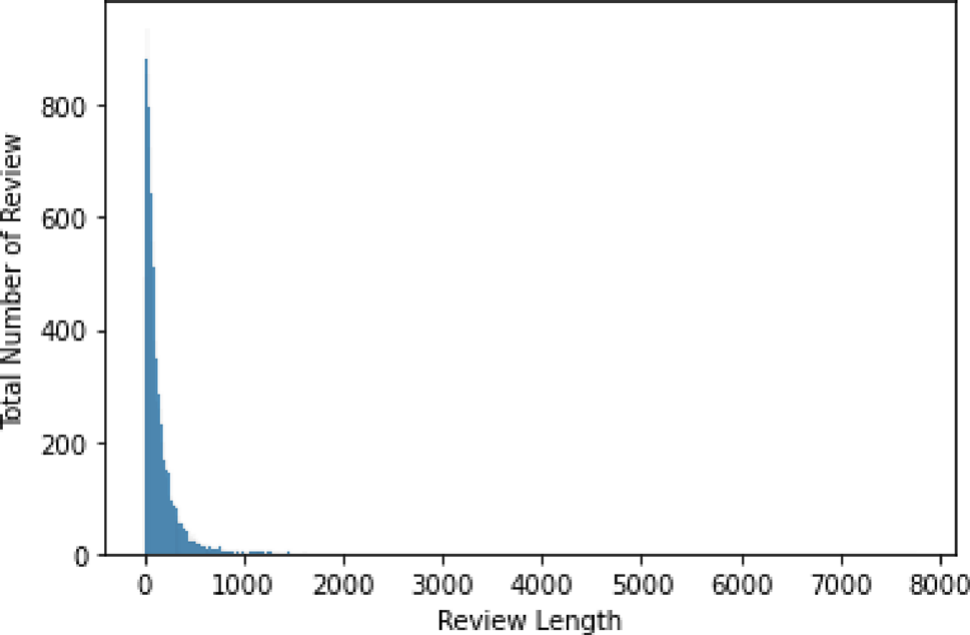
Distribution of token counts in review lengths.

- **Data preprocessing**: The dataset is pre-processed as follows for data preparation: precisely, we limit each review to 300 words (Fig. 3). The datasets are filtered to remove any courses with blank course descriptions. Figure 4 shows the distribution of token counts in review Lengths
- **Word embedding-based feature**: it involves employing the Glove technique to represent text data (posts, reviews, etc.) as real-valued N-dimensional vectors in a pre-defined vector space. Glove is a technique for unsupervised learning that extracts explicit data on word-to-word co-occurrence from text corpora^42,43^. Every review is converted into corresponding labels (numerical values) using the Glove embedding model for creating the vocabulary. These numerical values are used as input for the machine learning and advanced DRL will help to better understand the learners’ attitudes, behavior, and actions during the recommendations.
- **Data splitting**: For training and testing purposes, we divided the dataset in an 80:20 ratio for the training and testing model, respectively.

**Table 1:** Example of the course review dataset that is rated based on the given context.

ID	Course	Review	Context	Rate
510	Data-scientists-tools	A great introduction to Data Science and GitHub!	Sentiment (Satisfied)	5
112	Java-programming	It covers some basics but does not go deep into anything. Definitely take the other courses in the specialization to go deeper	Cognition	4
602	Machine-learning	Access to assignments and grades should be given	Cognition	3
115	R-PROGRAMMING	Great course for starters who are willing to pursue their career in R programming or Data Analysis	Sentiment (satisfied)	5
120	Python-data	This course does not contain any new information. It does not teach you but just excitedly shows commonly known facts. There are better ways to invest your time	Confusion	1

### Baseline models

To assess the accuracy of the proposed DRL models, we leveraged various baseline models which include a rating-based method matrix factorization (MF), multi-layer perceptron (MLP), SVD++, non-negative matrix factorization (NMF), and neural collaborative filtering (NCF). A detailed explanation of the baseline models is as follows:

**MLP**^44^ the recommendation probability is produced using an MLP on a pair of user and course embeddings.

**MF**^45^ is the commonly accepted baseline technique for CF-based RS. The recommendation algorithm is entirely rating-based and uses just rating data. In order to precisely examine the effects of using textual materials for RS, we employ this baseline methodology.

**NMF**^46^ represents a set of algorithms in the domain of multivariate analysis and linear algebra. By breaking a matrix down into non-negative factors, using NMF in RS enables better interpretability of the resulting components. When dealing with data like user-item interactions, where the lack of negativity is consistent with the underlying dynamics, this strategy is particularly helpful. This approach enables RS to efficiently identify patterns and latent features in the data, resulting in recommendations that are more precise and insightful.

**SVD++**^47^ is an extension of SVD considering implicit ratings such as learner and course bias. It adds a factor vector for each course, and these course factors are used to define the features of the course, regardless of whether it has been assessed. This introduces the implicit feedback information based on SVD.

**NCF**^44^ is a hybrid approach that integrates matrix factorization and MLP features for simulating learner-course latent frameworks, and it learns the likelihood of proposing target courses to associated learners.

**DQN**^48^ is a well-liked method for sequential decision-making problems. DQN can be used in RS to provide sequential recommendations when the sequence of the course is important. The suggestion of the next course is the action in this scenario, and the state could be the learners’ previous interactions or the current context. The suggestions are based on the actions with the greatest Q-values determined by the DQN model, which learns a Q-value function to predict the expected reward for each action.

### Parameter settings

A final dense layer with just one node is utilized to forecast the reward for the critic. The critic learns at a rate of 0.001, whereas the actor learns at a rate of 0.0001. Only 0.1% of the actors and critics are updated when the network is upgraded. We designed learning_rate = 0.001, *γ* = 0.99 (discount factor), epsilon = 0.1 (epsilon-greedy exploration factor). Based on the exploration probability, a student decides whether to explore or exploit. A course is picked at random if it opts to explore (based on a random number). Alternatively, depending on the Q-values (action probabilities), the actor framework estimates the probability of choosing each course. Moreover, a reward is determined for the student, following the course they selected and their interests. A reward also calculates whether the selected course aligns with the student’s interests. The proposed DRR model is subjected to rigorous training over 1000 episodes. Then, for each episode, a random student is chosen from the list of students.

### Evaluation metrics

The major evaluation metrics, including Hit Ratio (HR@N), Normalized Discounted Cumulative Gain (NDCG@N), recall@N, and precision@N^28,49^ are employed for evaluating the quality of recommendations. Where precision@N is the proportion of recommended items in the top-N set that are relevant (Eq. 4); HR@N is a recall-based metric that measures the percentage of the ground truth instances that are successfully recommended in top-N as determined in Eq. (5); recall@N denotes the percentage of preferred items in the top-N recommended list as denoted in Eq. (6); and NDCG@N assesses the position of a successful match in the recommendation list, assigning higher scores to hits at superior ranks within the list. The mathematical modelling of NDCG@N can be seen in Eq. (7). The top-N recommendations list is set as 5, 10, 15, and 20. We use the offline test method described in^49,50^ to evaluate the proposed models.

4 $$Precision@N= \frac{1}{\left|N\right|} \sum_{i=1}^{N}{r}_{i} \in \mathcal{T}$$

It can be determined that the model score for a given course j for student i is computed as the inner product.

5 $$HR@N = \frac{{\sum }_{u=1}^{U}{Hits}_{u}@N}{\left|GT\right|}$$

where ${Hits}_{u}@N$ stands for the number of courses in the top-N recommendation list for the u-th student pertaining to the testing set, |.| stands for the size of a set, and GT refers to the set of ground truths for all students in the testing set.

6 $$Recall@N = \frac{\left|\left\{r \in R : r\le N\right\}\right|}{|R|}$$

where R shows the total number of relevant courses for the given input. Precision@N and recall@N are binary metrics that can only be calculated for courses that a student has actually given a rating. The precision@N metric counts the proportion of generated recommendations that the student actually chose. Recall@N, on the other hand, gauges the proportion of all pertinent courses that the proposed model recommended.

7 $${NDCG}_{k}@N= \frac{{DCG}_{k}}{{IDCG}_{k}}$$

$${DCG}_{k}= \sum_{i=1}^{k}\frac{re{l}_{i}}{{log}_{2} (i+1)}$$

$${IDCG}_{k }= \sum_{i=1}^{k-ideal}\frac{{rel}_{i}}{{log}_{2} (i+1)}$$

where $re{l}_{i}u$ is the graded relevance of the recommendation result listed at position i to the u-th student, and IDCGk@N represents the ideal discounted cumulative gain obtained through the best top-N recommendation list for the u-th student.

### Experimental results, and analysis

Table 2 lists the overall performance of all the baselines and proposed methods. The proposed DRR model performs better than the comparison baselines in HR@10. The learner-to-content-based CF models, including MLP, NMF, MF, NCF, and SVD++ perform the worst among all the methods because, in the dataset, most of the learners participated in the review and only enrolled in a few courses. Thus, the embeddings for many students can not be sufficiently inferred from the sparse data. MF and NMF perform the worst of all the course-to-course-based CF algorithms because they consider all previously enrolled courses equally, which limits their ability to capture preferences. The effects of various previous courses are distinguished by MLP, NCF, and SVD++ by giving them various attention coefficients. When students enrol in numerous different courses, the effects of the useful courses in the past will be diminished. The proposed techniques, DRR, and DQN perform best because they compel the noisy courses to be removed.

**Table 2:** Recommendation performance of different methods measured by NDCG and HR using 100 K Coursera course reviews.

Models	HR (%)				NDCG (%)			
	5	10	15	20	5	10	15	20
MLP	53.44	64.54	67.25	70.06	50.65	51.84	53.51	54.37
NMF	46.71	49.66	52.98	57.77	43.54	46.98	48.68	50.01
SVD + +	48.87	50.01	53.33	54.50	43.65	47.09	50.12	52.45
MF	40.34	43.53	47.88	50.49	40.21	44.42	46.34	48.85
NCF	56.98	68.87	80.23	85.45	50.21	52.40	54.34	55.85
DQN	59.21	68.97	84.30	87.21	51.34	55.51	57.22	59.05
DRR	**64.12**	**75.64**	**86.11**	**93.52**	**57.47**	**60.03**	**51.66**	**64.26**

The highlighted results show the best performance.

Among the various CF models that are designed based on course-to-course interactions, MF and NMF tend to exhibit weaker performance compared to other models. This is due to their uniform treatment of all historically taken courses, which restricts their ability to accurately represent user preferences. On the other hand, models like MLP, NCF, and SVD++ with explicit feedback excel in distinguishing the impacts of different previously registered courses. They achieve this by assigning distinct attention coefficients to each course, thereby enhancing their predictive accuracy.

However, a challenge arises when students have enrolled in a multitude of diverse courses, causing less relevant courses to overshadow the effects of valuable ones from the past. To address this issue, the proposed approach DRR, as well as the DQN model, prove to be the most effective performers. These models adopt a strategy of deliberately eliminating irrelevant or noisy courses from consideration. This strategic course selection significantly enhances the overall predictive capability of these models and consequently leads to their superior performance.

Table 3 reports a comprehensive overview of the performance of both the baseline and the proposed approaches, where the best results are marked in bold type. Notably, the DRR model proposed in this study demonstrates superior performance compared to the baseline methods, specifically excelling in the recall@10 metric. It is important to highlight that the learner-to-course-based CF approaches, including MLP, NMF, MF, NCF, and SVD++, exhibit suboptimal performance. This is attributed to the dataset’s characteristics where most learners engage in providing reviews but enrol in only a limited number of courses. Consequently, the sparse data hinders the accurate inference of embeddings for many students.

**Table 3:** Recommendation performance of different methods measured by precision and recall using 100 K Coursera course reviews.

Models	Recall (%)				Precision (%)			
	5	10	15	20	5	10	15	20
MLP	54.21	65.04	68.10	71.02	53.54	56.30	58.51	60.13
NMF	47.00	50.43	53.56	58.42	55.65	58.12	59.98	61.23
SVD + +	40.34	43.53	47.88	50.49	48.21	54.42	57.34	58.85
MF	41.32	44.42	48.57	61.40	51.01	64.53	66.56	67.27
NCF	51.56	61.54	76.96	84.03	55.52	64.42	67.58	68.35
DQN	63.89	76.12	84.98	93.43	64.00	69.20	73.74	83.40
DRR	**65.01**	**76.53**	**88.11**	**94.02**	**68.12**	**71.24**	**79.99**	**85.31**

The best performance result is highlighted in bold.

Among the course-to-course-based CF models, both MF and NMF exhibit relatively weaker performance compared to their counterparts. This can be attributed to their uniform treatment of historically enrolled courses, limiting their ability to effectively represent preferences. In contrast, MLP, NCF, and SVD++ stand out by differentiating the impact of distinct historical courses through varying attention coefficients. Nevertheless, when users are enrolled in a diverse array of courses, the presence of less useful courses can dilute the effects of more valuable ones in their history.

**Figure 5:**
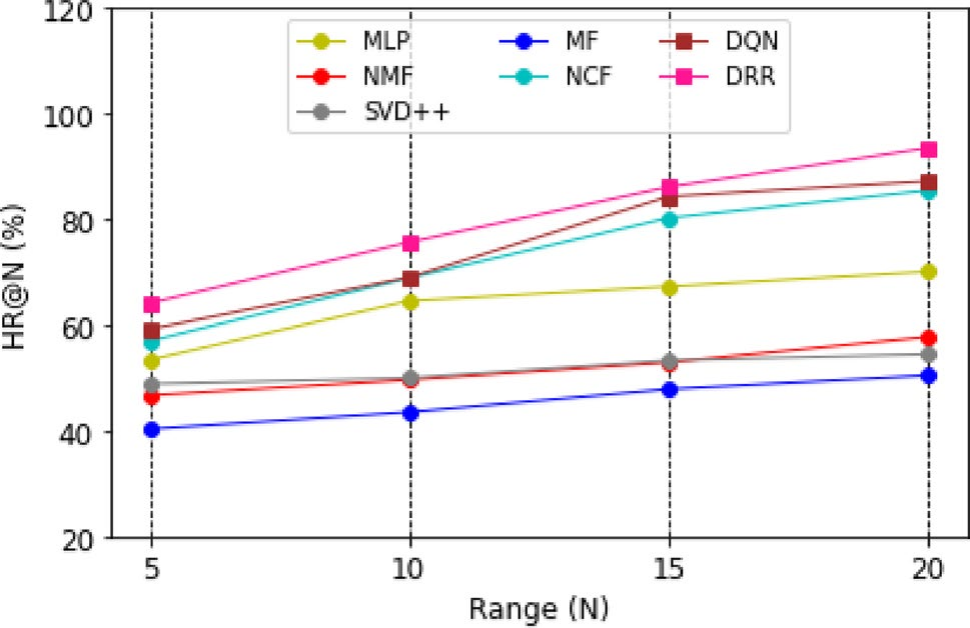
Evaluation index HR (%) for each model with the top (N = 5, 10, 15, 20).

**Figure 6:**
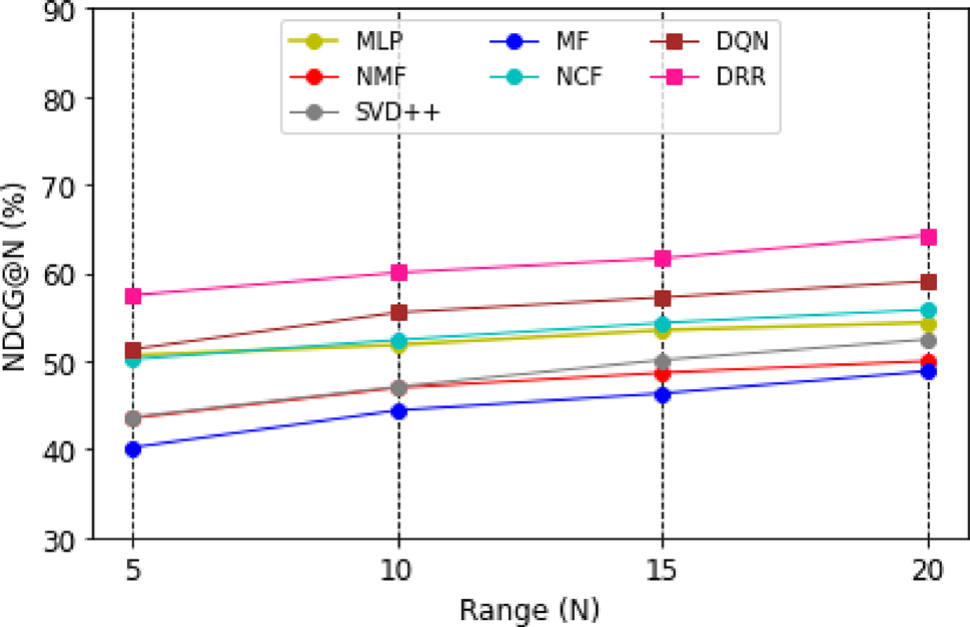
Evaluation index NDCG (%) for each model with top (N = 5, 10, 15, 20).

**Figure 7:**
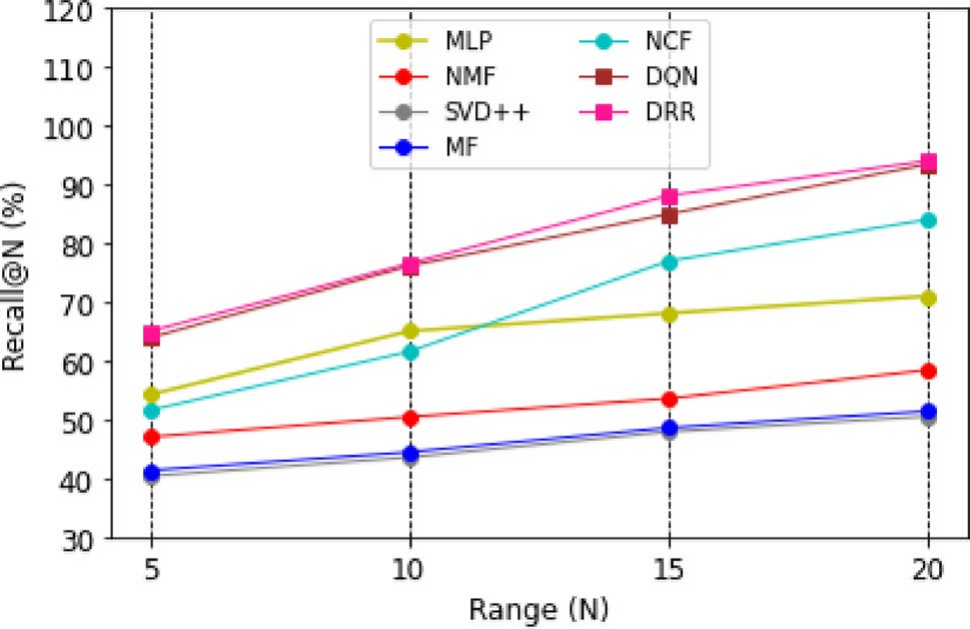
Evaluation index recall (%) for each model with a top (N = 5, 10, 15, 20).

**Figure 8:**
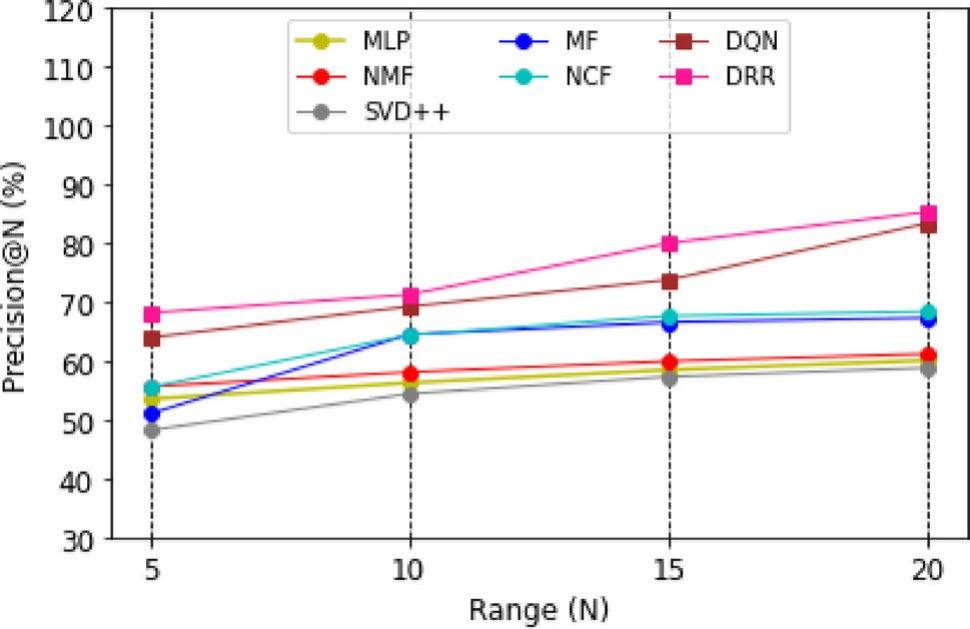
Evaluation index precision (%) for each model with the top (N = 5, 10, 15, 20).

Graphical representations of the experimental outcomes are depicted in Figs. 5, 6, 7 and 8. Figure 5 underscores the superior performance of the DRR model on the MOOC dataset in comparison to the baseline models, particularly evident in its higher HR@N metric. This highlights the model’s effectiveness. Besides, a comparison with the DQN model, which employs straightforward fully connected neural networks for state modeling, reveals a significant performance gap, showcasing the clear advantages of the proposed DRR model. The effectiveness of the DRR model is consistently demonstrated across multiple metrics, including NDCG@N, recall@N, and precision@N. These contrasts (NDCG@N, recall@N, and precision@N) are intensely presented in Figs. 6, 7 and 8, respectively.

Three conclusions can be drawn from the assessments. (1) Personalized adaptation and long-term planning are features of the suggested solutions that both traditional approaches and the NCF approach based on deep learning disregard. (2) Unlike DQN, which merely concatenates fully linked layers, the proposed state representation module effectively captures the complicated dynamic interactions between students and courses. This prevents information loss and subpar learning outcomes.

Likewise, compared with NCF, MF, MLP, NMF, and SVD +  + , it can be found that the DRR performs the best in terms of HR@N (Fig. 5), NDCG@N (Fig. 6), recall@N (Fig. 7), and precision@N (Fig. 8). The other performs less favorably. The reasons are as follows: the DRR outperforms because it captures not only the interactions between the learners’ historical courses but also the learner’s preferences for them. The experimental results demonstrate that the suggested DRR model has better recommendation performance than the other six models.

DRL-based methods use neural networks to approximate the value function and can handle high-dimensional and complex state spaces. These methods have been successfully applied to various problems and have demonstrated significant improvement in performance over traditional tabular CF-based RS methods. The integration of DRL and RS points towards a promising avenue for the development of more advanced and personalized recommendation systems.

## Conclusions

The significant growth in online learning platforms in recent years has revolutionized global education accessibility. Even though MOOCs let students complete their coursework, the abundance of course content available on the internet can make it challenging for certain learners to understand, especially those with low skills and cognitive abilities. The proposed solution to this problem is to use an adaptable and personalized RS integrated with an adaptive e-learning framework. Subsequently, a top-N MOOC course recommendation framework based on the DRR model is designed, trained, and evaluated using an Actor-Critic simulator. Through multiple iterations, a robust framework for training these models has been built, enabling the effective deployment of both DQN and Actor-Critic methodologies. The commonly used measures HR@N, Recall@k, Precision@k, and NDCG@N are used to evaluate each approach. The findings of this study demonstrate how new methods based on DRL can be used to recommend top-N courses in MOCC. Both DQN and DRR can reach the goal by suggesting the top-N courses, although DRR (Actor-Critic) obtains better performance and recommendations are more personalized to the learners’ preferences and interests. Our suggested framework, which is based on DRL for top-N course recommendations, is intended to act as a strong baseline for RSs in MOOCs. This framework has been rigorously evaluated within practical simulated environments, solidifying its contribution to the online learning domain.

### Implications

The DRR framework is designed to intelligently assess students’ acceptance of technology and well-being while recommending relevant learning content. It enables continuous learning by adapting recommended courses to changing learner interests, ensuring an adaptable and personalized system. This streamlined learning process benefits both students and teachers. The proposed adaptable and personalized DRR model has the potential to significantly impact e-learning technology, improving the design and execution of course RS. In order to support students’ interests and preferences, it also automates administrative tasks and helps teachers better understand the unique needs of each student.

In addition, the enhanced adaptable and personalized RS framework for introducing online course content is not limited to academic course recommendations. It can also be employed for efficient information retrieval on e-commerce and news websites, providing suggestions for things like headlines on news sites or products on Amazon, Ali Express, etc.

### Limitations and future work

One of the foremost challenges encountered during the exploration of this subject was the identification of suitable learning environments. The availability of open-source environments was limited. In certain RS, the signal for rewards is weak. As a result, the agent might not get paid for many actions. The agent may find it challenging to understand the best course of action as a result.

The study has many motivations for future research developments that we will explore can be: in the future, we aim to extend this study by incorporating multiple MOOC datasets encompassing a larger number of students and courses. The work can be enhanced further by using other DRL techniques like Double DQN, Dueling DQN, and Deep Deterministic Policy Gradient (DDPG). We also aim to evaluate the effectiveness of various approaches for top-N courses and quantify their effects on recommendation quality. This comprehensive assessment will be carried out through experiments conducted in both offline and simulated online evaluation contexts.

## Data Availability

The datasets generated and/or analysed during the current study are available in the Kaggle repository https://www.kaggle.com/datasets/septa97/100k-courseras-course-reviews-dataset.
